# Redox Imbalance in Gestational Diabetes Mellitus: Mechanistic Insights, Emerging Biomarkers, and Therapeutic Perspectives

**DOI:** 10.3390/ijms27114755

**Published:** 2026-05-25

**Authors:** Chinnappa A. Uthaiah, Tarun Sahu, Vinita Singh, Jessy Abraham

**Affiliations:** 1 Department of Biochemistry, All India Institute of Medical Sciences (AIIMS), Raipur 492099, Chhattisgarh, India; auchinnappa16@gmail.com; 2 Genetics Laboratory, All India Institute of Medical Sciences (AIIMS), Raipur 492099, Chhattisgarh, India; 3 Department of Physiology, All India Institute of Medical Sciences (AIIMS), Raipur 492099, Chhattisgarh, India; 4 Department of Obstetrics and Gynaecology, All India Institute of Medical Sciences (AIIMS), Raipur 492099, Chhattisgarh, India; ddvinitasingh@gmail.com

**Keywords:** gestational diabetes mellitus, oxidative stress, inflammation, signaling pathways

## Abstract

Gestational diabetes mellitus (GDM) is increasingly recognized as a complex pathology rooted in systemic and organelle-level dysfunction, specifically involving chronic low-grade inflammation (CLGI), mitochondrial impairment, and endoplasmic reticulum (ER) stress. Central to this pathophysiology is mitochondrial dysfunction, characterized by reduced respiration, impaired metabolic flexibility, and dysregulated fission/fusion machinery, which fuels a self-perpetuating cycle of reactive oxygen species (ROS) production. Concurrently, chronic ER stress triggered by hyperglycemia and lipotoxicity activates the unfolded protein response (UPR), further amplifying redox imbalance through the Endoplasmic Reticulum Oxidoreductin 1/Protein Disulfide Isomerase (ERO1/PDI) axis and bridging metabolic toxicity to inflammation via c-Jun N-terminal kinase (JNK) and nuclear factor kappa-light-chain–enhancer of activated B cells (NF-κB) signaling. The Advanced Glycation Endproducts (AGEs) and the Receptor for Advanced Glycation Endproducts (RAGE) axis act as a molecular catalyst that sequester antioxidants and drive pro-inflammatory feedback loops. These converging mechanisms culminate in profound placental maladaptation, including structural abnormalities like chorangiosis and functional defects in nutrient transport mediated by hyperactive mechanistic target of rapamycin complex 1 (mTORC1) signaling. This review article provides insight into recent evidence to elucidate the meta-inflammatory environment of GDM, where modest but sustained elevations in biomarkers like Interleukin-6 (IL-6) and tumor necrosis factor-alpha (TNF-α) disrupt redox homeostasis and impair insulin signaling pathways through the activation of stress-sensitive kinases. By integrating these molecular perspectives, the article underscores the necessity of targeting the systemic inflammatory and oxidative continuum spanning pre-conception to the antenatal period through lifestyle interventions and emerging therapeutic strategies to mitigate GDM risk and improve maternal–fetal outcomes.

## 1. Introduction

GDM refers to the glucose intolerance first identified in the second or third trimester in pregnancy in individuals who did not have diabetes before becoming pregnant [1]. The International Diabetes Federation estimates that approximately 23.3 million pregnancies are affected by hyperglycemia during pregnancy, the majority of which are attributed to GDM. [2]. Using uniform criteria, the global prevalence in 2021 was estimated at 14%. This figure varies significantly by region, with the highest rates in the Middle East and North Africa (27.6%) and South-East Asia (20.8%) and the lowest in North America and the Caribbean (7.1%) and Europe (7.8%) [3]. In developing countries like India, its prevalence ranges from 4.2% to 13% which reflects significant regional disparities driven by genetic predisposition, dietary patterns, and diagnostic criteria employed [4,5]. GDM presents a wide range of clinical effects in both the mother and the child. It raises the future probability of developing type 2 diabetes and cardiovascular disease in the mother, whereas it can lead to macrosomia, neonatal hypoglycemia, and respiratory distress in infants. In addition, long-term complications in offspring exposed to GDM include higher risks of obesity, type 2 diabetes, and cognitive or developmental challenges [6]. Beyond glycemic dysregulation, GDM substantially elevates maternal risk for a group of cardiovascular diseases, such as coronary artery disease, myocardial infarction, ischemic stroke, heart failure, and atrial fibrillation [7]. It also carries immediate obstetric concerns, as GDM is associated with increased rates of preeclampsia, cesarean delivery, preterm birth, and maternal hypertensive disorders [8].

Redox imbalance, characterized by an excess of ROS over antioxidant defenses (oxidative stress) is a critical factor in the pathogenesis of GDM. It is a critical mediator that links hyperglycemia to cellular dysfunction, insulin resistance, and tissue damage in pregnancy [9]. Physiological pregnancy is characterized by increased oxidative metabolism, with the placenta serving as the primary source of ROS during gestation. Under normal circumstances, this elevation in ROS is counterbalanced by enhanced synthesis of endogenous antioxidants and maintains the redox homeostasis, which is essential for placental development and fetal growth [10]. However, in GDM, this delicate equilibrium is profoundly disrupted. The aim of the present review is to assess the role of redox imbalance in the pathophysiology of GDM. We tried to explore mechanistic insights and highlight the emerging redox imbalance biomarkers with diagnostic potential. Further, we also examine the therapeutic strategies to counteract the redox imbalances. By focusing on redox imbalance independently of inflammatory mechanisms, this review explains a targeted framework for understanding redox biology in GDM.

## 2. Mechanisms Connecting Redox Imbalance to GDM

**Figure 1:**
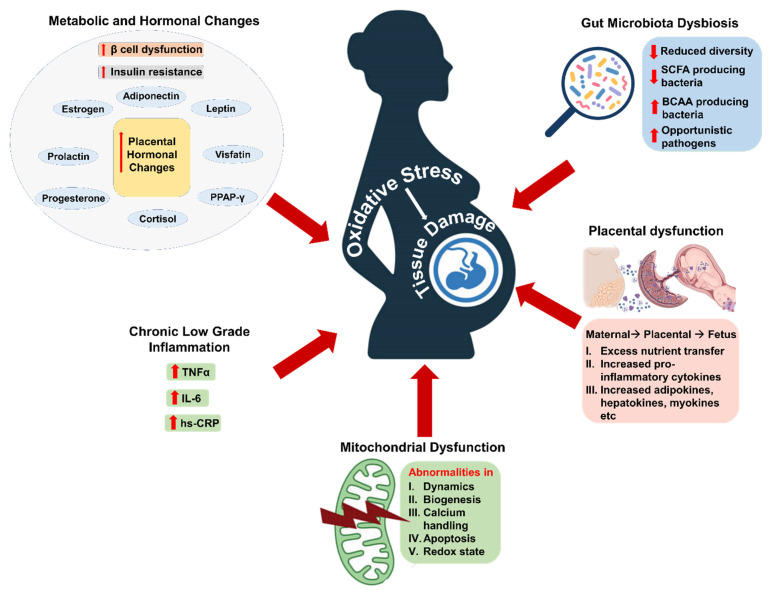
Mechanisms responsible for inducing oxidative stress during GDM. A schematic illustration representing the metabolic and hormonal changes, chronic low-grade inflammation, mitochondrial dysfunction, placental dysfunction, and gut microbiota dysbiosis as major causative factors for the induction of oxidative stress during pregnancy. TNF-α: Tumor necrosis factor alpha; IL-6: Interleukin-6; hs-CRP: highly sensitive C-reactive protein; SCFA: Shor-chain fatty acid; BCAA: branched-chain amino acid.

The pathogenesis of GDM is driven by a complex interplay of redox imbalance and systemic metabolic disturbances. CLGI initiates this cascade, where pro-inflammatory cytokines activate stress-sensitive kinases that impair insulin signaling. This meta-inflammatory state is exacerbated by mitochondrial dysfunction and ER stress, which creates ROS production. Furthermore, AGEs and gut microbiota dysbiosis contribute to the oxidative continuum. These mechanisms converge to induce the placental dysfunction characterized by the impaired nutrient supply and increased stress on the fetus as shown in Figure 1.

### 2.1. Metabolic and Hormonal Shifts in Pregnancy

Normal pregnancy involves major metabolic shifts that naturally create a more oxidative environment, which in the context of GDM becomes markedly exaggerated. Pregnancy progresses through two different metabolic phases: the anabolic phase, which is focused on maternal nutrient storage, and the catabolic phase, which is dedicated to fetal growth. During the catabolic phase (in the second and third trimesters), maternal insulin sensitivity declines to around 50% of that of the pregestational state, which necessitates a compensatory 200–250% increase in insulin secretion to maintain euglycemia [11,12,13,14]. This physiological insulin resistance is determined by multiple placental hormones that reprogram the metabolism during the catabolic stage. Human placental lactogen, secreted from the sixth week of gestation and reaching peak concentrations around 30 weeks, exerts potent diabetogenic effects by promoting lipolysis, increasing circulating free fatty acids, and antagonizing insulin action [11,15]. Placental growth hormone, secreted tonically rather than pulsatilely, induces hyperinsulinemia, decreases insulin-stimulated uptake of glucose and glycogen synthesis, and impairs the ability of insulin to suppress hepatic gluconeogenesis. Progesterone suppresses the phosphatidylinositol 3-kinase (PI3K) pathway by reducing expression of insulin receptor substrate-1 (IRS-1), thereby inhibiting insulin-induced glucose transporter-4 (GLUT4) translocation. Additionally, elevated cortisol levels resulting from increased placental adrenocorticotropic hormone secretion, along with inflammatory cytokines such as TNF-α and IL-6, further interrupt insulin signaling pathways [11,12,16].

Recent transcriptomic studies have identified insulin-like growth factor binding protein-1(IGFBP-1) as a critical placental factor positively associated with insulin sensitivity, with low circulating levels in early pregnancy predicting subsequent GDM diagnosis. When the maternal pancreatic β-cells fail to adequately compensate for pregnancy-induced insulin resistance, either due to pre-existing β-cell dysfunction or inadequate adaptive proliferation, hyperglycemia develops. Women with obesity or a history of GDM enter pregnancy with pre-existing insulin resistance that worsens with advancing gestation and demonstrate only 80% suppression of hepatic glucose production compared to 95% suppression in healthy pregnant women [12,17,18].

The metabolic shift toward lipid metabolism in late pregnancy also creates an additional pro-oxidative condition. During the third trimester, lipoprotein lipase activity increases, lipolysis from adipose tissue accelerates, and free fatty acid levels rise substantially, making lipids the key maternal energy source, whereas glucose and amino acids are preferentially reserved for the fetus. But in GDM, hyperglycemia induces increased placental lipoprotein lipase activity, which enhances the hydrolysis of maternal lipoproteins for transport across the placenta. Recent studies have positively correlated both maternal triglycerides and non-esterified fatty acids with neonatal adiposity [12,19]. These metabolic imbalances converge and give rise to the state of increased oxidative stress. The placenta serves as the primary source of ROS and shows markedly elevated oxidative metabolism in GDM. Under normal circumstances, the physiological increase in oxidative stress is counterbalanced by higher synthesis of endogenous antioxidants, but in GDM, this equilibrium is profoundly disrupted, resulting in devastating oxidative damage to placental tissues, maternal circulation, and ultimately fetal tissues [10,14].

### 2.2. Chronic Low-Grade Inflammation

GDM is characterized by increased levels of pro-inflammatory cytokines like TNF-α and IL-6, secreted by adipose tissue and the placenta. These mediators interfere with insulin signaling by promoting serine phosphorylation of insulin receptor substrates, which ultimately exacerbates insulin resistance and leads to the production of more ROS [20,21,22,23].

### 2.3. Mitochondrial Dysfunction

Hyperglycemia and hyperlipidemia in GDM lead to increased production of ROS within the mitochondria, the main source of cellular energy. This dysfunction impairs the normal function of the electron transport chain, reduces ATP production, and triggers pro-inflammatory pathways, further contributing to β-cell damage and insulin resistance [24,25,26].

### 2.4. Endoplasmic Reticulum (ER) Stress

The prolonged demand on pancreatic β-cells to produce excessive insulin, coupled with chronic inflammation, can induce ER stress. This stress leads to increased β-cell apoptosis (cell death) and reduced insulin gene expression, contributing to β-cell failure [12,27,28].

### 2.5. Advanced Glycation Endproduct Formation

In a hyperglycemic environment, glucose non-enzymatically reacts with proteins and lipids to form AGEs. The binding of AGEs to RAGE activates transcription factors like NF-κb and stimulates ROS formation through NADPH oxidase (NOX), which promotes oxidative stress and inflammation, damaging endothelial cells and impairing insulin signaling [29,30].

### 2.6. Placental Dysfunction

**Figure 2:**
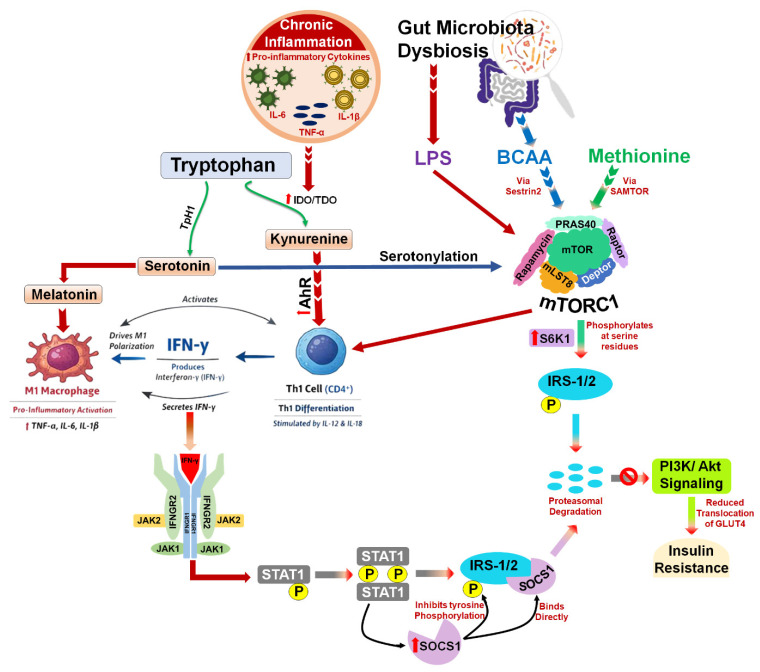
Integrated mechanistic overview of inflammatory signaling in the pathogenesis of insulin resistance in GDM. The schematic illustrates the interplay between chronic inflammation, gut microbiota dysbiosis, and amino-acid-mediated signaling pathways contributing to insulin resistance in GDM. IL-6: Interleukin 1 beta; TNF-α: tumor necrosis factor alpha; IDO: Indoleamine 2,3-dioxygenase; TDO: tryptophan 2,3-dioxygenase; TpH1: Tryptophan Hydroxylase 1; IFN-γ: interferon gamma; Th1:Type 1 Helper T cells; IFNGR: interferon gamma receptor; JAK: Janus Kinase; STAT: Signal Transducer and Activator of Transcription; SOCS: Suppressor of Cytokine Signaling; IRS: insulin receptor substrate; AhR: Aryl hydrocarbon Receptor; S6K1:Ribosomal protein S6 kinase beta-1; LPS: lipopolysaccharide; BCAA: branched chain amino acids; PRAS40: Proline-rich Akt Substrate of 40 kDa; mTOR: Mechanistic Target of Rapamycin; PI3K: Phosphoinositide 3-kinase.

The placenta plays a central role as an endocrine organ, but in GDM, it exhibits increased oxidative stress and altered expression of adipokines and cytokines. This local inflammation and oxidative damage impair placental vascular function and nutrient transport, contributing to maternal metabolic disturbances and fetal overgrowth [31,32,33,34]. Elevated Interferon-gamma (IFN-γ) produced by T helper 1 (Th1) cells also shifts the M1/M2 macrophage equilibrium in the placenta. The placental macrophages exhibit phenotypic plasticity, enabling them to transition between pro-inflammatory M1 and anti-inflammatory M2 states under different microenvironment conditions [35]. In a recent study, Barbosa et al. demonstrated that maternal hyperglycemia alters placental cytokine profiles and macrophage polarization patterns, highlighting the dynamic balance between M1 and M2 macrophages at the maternal–fetal interface [36]. IFN-γ activates the Janus Kinase 1/Signal Transducer and activator of transcription 1 (JAK1/STAT1) pathway in macrophages, inducing the expression of pro-inflammatory genes and suppressing M2-associated markers such as Arginase-1, Interleukin-10 (IL-10), and Cluster of Differentiation 206 (CD206), which collectively drives M2 to M1 polarization [37]. The increased population of M1 macrophages subsequently releases high levels of pro-inflammatory cytokines, including TNF-α, IL-6, and Interleukin-1 Beta (IL-1β) [38]. These cytokines activate stress-related kinases such as Jnk and inhibitor of κb Kinaseβ (Ikkβ), leading to phosphorylation of serine residues in IRS-1 rather than the physiologically required tyrosine phosphorylation [39]. This modification generates a binding site for the skp1-Cullin-F-Box (Protein) Β-transducin repeat-containing protein (SCF^β TRCP) E3 ubiquitin ligase, promoting IRS-1 ubiquitination and subsequent proteasomal degradation [40]. This reduces the pool of active IRS receptors that can interact with the insulin receptor. Consequently, PI3K recruitment and phosphatidylinositol (3,4,5)-trisphosphate (PIP3) synthesis at the plasma membrane are impaired, which diminishes downstream Rac-Alpha Serine/threonine-Protein Kinase (AKT) activation and the translocation of GLUT4 to the plasma membrane, markedly reducing glucose uptake in adipose tissue, skeletal muscle, and liver [41,42]. In addition, IL-6 secreted by M1 macrophages further exacerbates insulin resistance by inducing suppressor of cytokine signaling-3 (SOCS3) expression [43]. SOCS3 directly interacts with the insulin receptor and IRS proteins, inhibiting receptor tyrosine kinase activity and promoting degradation of insulin signaling components [44]. M1 macrophages also produce Interleukin-12 (IL-12), which promotes Th1 differentiation and sustained IFN-γ production [45]. This feed-forward loop not only reinforces the pro-inflammatory environment at the maternal–fetal interface but also exacerbates insulin resistance by impairing IRS-1 Signaling, and GLUT4-mediated glucose uptake in maternal tissues. An illustrative overview of the signaling pathway is presented in Figure 2.

### 2.7. Epigenetics and Nutrigenomics in GDM

Genetic predispositions and epigenetic modifications (changes in gene expression without altering the DNA sequence, such as DNA methylation and altered micro-RNA levels) influence an individual’s susceptibility to GDM. Nutrigenomics refers to the interaction between dietary components and the genome, influencing gene expression, epigenetic programming, and metabolic phenotypes. These factors can affect pancreatic β-cell function, insulin sensitivity, and inflammatory responses, with some alterations potentially persisting long after pregnancy and even across generations [46,47].

Folate and methionine are critical regulators of one-carbon metabolism, a central biochemical pathway that sustains DNA methylation and normal gene expression by facilitating the transfer of one-carbon units required for DNA synthesis, methylation reactions (via S-adenosylmethionine, (SAM)), and cellular redox balance. When SAM levels are high, SAM binds to the protein S-adenosylmethionine sensor upstream of mTORC1 (SAMTOR), causing it to dissociate from the GTPase-Activating Transferase operating toward Rags 1 (GATOR1)–Kaptin Integrin alpha-FG-GAP repeat-containing protein 2 C12 or f66 seizure threshold 2 Target of Rapamycin (KICSTOR) complex. This dissociation prevents GATOR1 from inhibiting mTORC1, thereby allowing mTORC1 to be activated and subsequently activate ribosomal protein S6 kinase beta-1 (S6K1) [48,49].

Vitamin B12 acts as an essential cofactor for methionine synthase, which converts homocysteine to methionine using 5-methyltetrahydrofolate (5-methylTHF) derived from folate. Vitamin B12 deficiency therefore, leads to the “methyl-folate trap,” resulting in functional folate deficiency and elevated homocysteine levels. Elevated homocysteine, in turn, disrupts insulin signaling through both receptor-level and intracellular inflammatory mechanisms. At the ER level, homocysteine impairs proper folding and maturation of the pro–insulin receptor, reducing the availability of functional insulin receptors at the cell surface and thereby decreasing insulin sensitivity [50]. In parallel, it activates stress-responsive kinases, particularly JNK, along with NF-κB and Activator Protein-1 (AP-1), which promote transcription of pro-inflammatory mediators such as TNF-α and Monocyte Chemoattractant Protein-1 (MCP-1) and sustain CLGI. Activated JNK further impairs insulin signaling by phosphorylating IRS-1 at inhibitory serine residues, thereby blocking downstream insulin receptor signaling [51].

Polyunsaturated fatty acids (PUFAs) and their derivatives act as natural ligands for Peroxisome Proliferator-Activated Receptors (PPARs), which are transcription factors that regulate fatty acid oxidation and storage [52]. PUFAs suppress lipogenic genes by reducing the expression of transcription factors like Sterol Regulatory Element-Binding Protein-1c (SREBP-1c), which are responsible for lipid synthesis (lipogenesis) and upregulating genes involved in fatty acid oxidation (e.g., Carnitine Palmitoyltransferase I (CPT-1)) [53]. PUFAs also activate membrane receptors like G Protein-Coupled Receptor 120 (GPR120) (commonly known as Free Fatty Acid Receptor 4 (FFAR4)) and G Protein-Coupled Receptor 40 (GPR40), which are highly expressed in pancreatic beta-cells and adipocytes [54]. This activation promotes anti-inflammatory effects and enhances glucose-induced insulin secretion, mitigating the hyperglycemia of GDM.

Additionally, the maternal diet influences gut microbiota composition, generating metabolites such as short-chain fatty acids (SCFAs) that function as histone deacetylase inhibitors and modulate gene transcription in metabolic and inflammatory pathways [55]. Through this integrated nutrigenomic–epigenetic network, the maternal diet and metabolic environment can reshape gene expression profiles that contribute to insulin resistance and altered placental nutrient transport in GDM while also potentially programming long-term metabolic susceptibility in the offspring.

### 2.8. Gut Microbiome Dysbiosis

Alterations in the composition of the gut microbiota observed in women with GDM can influence host metabolism, exacerbate inflammation and oxidative stress, reduce insulin sensitivity, and impact the initial gut ecosystem of the offspring [56,57]. Emerging evidence highlights the placenta gut microbiota axis as a critical regulator of maternal and fetal metabolic homeostasis. Dysbiosis in pregnancy, an imbalance in the gut microbiome, is primarily caused by metabolic changes (obesity, diabetes), poor diet (high fat, low fiber), and high stress levels, among other contributors. The gut microbiota comprises a diverse community of microorganisms, including bacteria, viruses, and fungi, residing in the gastrointestinal tract. These microbes exist in a symbiotic relationship with the host and play a key role in maintaining physiological homeostasis, particularly through regulation of immune function and modulation of nutrient metabolism and energy balance [58]. *Faecalibacterium prausnitzii* and other butyrate-producing bacterial strains encode for branched-chain amino acid (BCAA) transporters that are involved in the uptake of BCAAs, acting as a “Sink” that reduces the availability of BCAAs for systemic absorption [59]. Dysbiosis During Pregnancy Is Frequently Characterized by A Reduction in Beneficial Commensals, Such as *Faecalibacterium prausnitzii*, along with an increase in BCAA-producing bacteria, including *Prevotella copri* and *Bacteroides vulgatus*, which are associated with elevated circulating BCAA levels [60]. Elevated BCAAs, as discussed in the context of amino acid metabolism, can impair insulin sensitivity by activating the mTOR signaling pathway, thereby disrupting insulin signaling in key metabolic tissues.

A decline in *Faecalibacterium prausnitzii* and other SCFA-producing bacteria also results in decreased expression of tight junction protein (e.g., occludin, claudins, Zonula Occludens-1 (ZO-1)), leading to increased paracellular permeability [61]. There is reduced mucus secretion by goblet cells, which diminishes the protective layer that normally limits microbial translocation [62]. This leads to compromised intestinal barrier integrity, allowing bacterial lipopolysaccharide (LPS) produced by Gram-negative bacteria to enter the circulation. Once systemic, free LPS activates toll-like receptor 4 (TLR4) signaling through the formation of a multiprotein receptor complex. LPS is first bound by LPS-binding protein (LBP) and transferred to Cluster of Differentiation 14 (CD14), which presents it to the TLR4–Myeloid Differentiation Factor 2 (MD-2) complex. Binding induces TLR4/MD-2 dimerization, enabling recruitment of adaptor proteins Myeloid Differentiation Primary Response 88 (MyD88) and TIR-domain-containing adapter-inducing interferon-β (TRIF). These pathways activate downstream kinases, including JNK and IKK, which phosphorylate serine residues on IRS proteins [63]. In addition, SCFAs produced by gut microbiota, including acetate, propionate, and butyrate, can activate G-protein-coupled receptors such as GPR41 (FFAR3) and GPR43 (FFAR2) on adipocytes, immune cells, and intestinal epithelial cells [64]. Activation of these receptors triggers intracellular signaling cascades, including Mitogen-Activated Protein Kinase (MAPK), NF-κB, and JNK pathways, with JNK-mediated serine phosphorylation of IRS-1 [65,66]. Serine-phosphorylated IRS is targeted for ubiquitin-mediated proteasomal degradation, reducing IRS availability and impairing downstream PI3K-AKT signaling. This disruption diminishes GLUT4 translocation in muscle and adipose tissue and impairs hepatic insulin signaling. Collectively, these mechanisms illustrate the placenta–gut microbiota axis, in which microbial metabolites and components converge on stress kinase pathways in both maternal and placental tissues, disrupting insulin signaling and potentially influencing fetal metabolic programming.

## 3. Primary Mechanisms Driving Redox Imbalance in GDM

### 3.1. Mitochondrial Dysfunction and ROS Generation

Hyperglycemia-induced mitochondrial dysfunction is a major source of ROS in GDM [67]. When cellular energy metabolism exceeds a critical limit, the normal flow of electrons through the mitochondrial electron transport chain becomes disrupted, particularly at Complex III. Under these conditions, electrons accumulate in coenzyme Q (ubiquinone) and may prematurely react with oxygen at the Qo site, leading to the formation of superoxide radicals. This abnormal electron leakage is a major source of oxidative stress within mitochondria [14,68,69]. This process is further amplified by the adaptor protein p66Shc (66 kDa Src homology-collagen homolog), which promotes the diversion of electrons from the coenzyme Q pool toward oxygen, thereby increasing mitochondrial reactive oxygen species (ROS) production. Elevated ROS levels subsequently activate dynamin-related protein 1 (Drp1), a key regulator of mitochondrial fission, resulting in excessive mitochondrial fragmentation [70]. In the context of GDM, increased expression of both p66Shc and Drp1 has been reported in placental tissues, indicating enhanced oxidative stress and mitochondrial dysfunction. Under persistent hyperglycemic conditions, the expression of these proteins rises progressively, reflecting cumulative mitochondrial damage as the disease advances. This is accompanied by structural alterations, including disruption of mitochondrial cristae and loss of membrane integrity [70,71].

Reduced import of Coenzyme A (CoA) limits the availability of intermediates required for the TCA cycle and oxidative phosphorylation. A reduction in inner mitochondrial membrane (IMM) translocase proteins leads to a deficiency in functional components of the electron transport chain. This disrupts the flow of electrons and the leakage of electrons to molecular oxygen.

Further, hyperglycemia reduces the expression of the CoA importer (SLC25A42) and IMM translocases in pancreatic β-cells. This leads to diminished mitochondrial bioenergetic capacity, resulting in reduced Adenosine Triphosphate (ATP) production from glucose and, consequently, impaired ATP-dependent insulin secretion in pancreatic β-cells, contributing to β-cell dysfunction [67,72,73,74].

### 3.2. Polyol-Pathway-Driven Redox Imbalance

The polyol pathway is increasingly recognized as a hyperglycemia-responsive metabolic route that, through excess glucose shunting, contributes to redox imbalance in GDM. Under normoglycemic conditions, less than 3% of glucose enters this pathway; however, during hyperglycemic conditions, about 30% of cellular glucose is metabolized via this route [75]. The pathway itself consists of two sequential enzyme reactions: aldose reductase, which reduces glucose to sorbitol using NADPH as a cofactor, and then sorbitol dehydrogenase, which oxidizes sorbitol to fructose using NAD as a cofactor, thereby generating NADH. The consumption of NADPH by aldose reductase depletes this critical cofactor required by glutathione reductase for regenerating reduced glutathione, the cell’s primary antioxidant defense molecule. This redox imbalance also compromises the activity of NADPH-dependent enzymes, such as nitric oxide (NO) synthase and cytochrome P450, which lead to impaired endothelial function. Simultaneously, the oxidation of sorbitol produces excess NADH, which creates a redox imbalance by an elevated NADH/NAD^+^. Additionally, sorbitol accumulation within cells creates osmotic stress that further exacerbates cellular dysfunction [75,76].

Activation of the polyol pathway also activates and amplifies multiple mechanisms of cellular damage, such as protein kinase C (PKC) activation, AGE formation, oxidative and nitrosative stress, and poly (ADP-ribose) polymerase (PARP) activation [77]. Glucose metabolism through the glycolytic pathway produces diacylglycerol (DAG) as one of the intermediates. DAG activates PKC, a central regulator of cellular signaling. Hyperglycemia enhances glycolysis and subsequently increases DAG production, leading to sustained PKC activation in multiple tissues, including vascular endothelium, smooth muscle, and placental trophoblasts [78,79]. PKC, in turn, stimulates ROS generation by activating enzymes such as NOX and impairs antioxidant defense mechanisms, thereby intensifying oxidative stress. In vascular endothelial cells, PKC directly phosphorylates endothelial nitric oxide synthase (eNOS) at the inhibitory threonine 495 residue while simultaneously promoting dephosphorylation of the activating serine 1177 site, resulting in eNOS inactivation. Chronic PKC activation, as observed under hyperglycemic conditions, also decreases eNOS gene expression, reducing the overall availability of the enzyme for NO production. Simultaneously, PKC activation of NOX drives excessive superoxide generation, which rapidly reacts with the diminished NO pool to form peroxynitrite (ONOO^−^), a highly potent oxidant that induces cellular damage and further amplifies PKC signaling, establishing a vicious cycle of oxidative stress [78,79].

### 3.3. NF-κb–Mediated Inflammatory Redox Dysregulation

Persistent hyperglycemia orchestrates a complex feedback loop in which inflammation; NF-κB, an essential regulator of inflammatory gene expression; and redox imbalance are interconnected through a complex feedback loop that mutually amplifies their pathogenic effects [80]. Hyperglycemia-induced ROS act as intracellular signals that directly upregulate Toll-like receptor (TLR) expression and activity, leading to activation of the IκB kinase (IKK) complex. IKK phosphorylates IκB proteins, promoting IκBα degradation and thereby releasing NF-κB (p50/p65) to translocate into the nucleus. Nuclear NF-κB drives the transcription of pro-inflammatory cytokines, including TNF-α, IL-1β, and IL-6, as well as key pro-oxidant enzymes such as inducible nitric oxide synthase (iNOS), which generates high levels of NO, and NOX, a major source of superoxide radicals [81,82,83]. The resulting ROS and reactive nitrogen species (RNS), particularly hydrogen peroxide (H_2_O_2_), further enhance NF-κB signaling by oxidatively modifying cellular proteins such as Dynein Light Chain 8 (LC8), facilitating its dissociation from IκBα and enabling sustained IKK-mediated NF-κB activation [84].

Hyperglycemia stimulates A Disintegrin and Metalloproteinase 17 (ADAM17), also known as TNF-α Converting Enzyme (TACE), which cleaves membrane-bound TNF-α, releasing its soluble form into the extracellular space. The liberated TNF-α binds to TNF receptors (TNFRs), ubiquitously expressed on nearly all cell types, triggering the IKK complex and thereby amplifying NF-κB activation [85,86]. NF-κB activation contributes to mitochondrial dysfunction, increasing the production of ROS within the mitochondria, further adding to the oxidative stress burden. The placenta acts as a source of inflammation, with GDM placentas showing increased expression of NF-κB and related pathways, particularly when associated with maternal obesity [87]. Research indicates that histone modifications, specifically Mixed-Lineage Leukemia 1 (MLL1)-dependent Histone H3 Lysine 4 Trimethylation (H3K4me3), can upregulate the NF-κB p65 subunit promoter in GDM, further driving the inflammatory response [88].

High glucose stimulates macrophages to secrete IL-1β through mechanisms involving inflammasome activation and NF-κB pathway engagement, which in turn increases NOX activity and amplifies NF-κB signaling, leading to elevated ROS production. IL-1β signaling also induces ER stress and the UPR, which generates additional ROS via oxidative protein folding and exacerbates oxidative stress within the cell. ER stress and ROS further activate stress kinases such as JNK and IKKβ, impairing insulin signaling by promoting inhibitory serine phosphorylation of IRS-1 and reinforcing inflammatory NF-κB activity, thereby contributing to insulin resistance. Moreover, IL-6 trans-signaling drives the expression of suppressors of cytokine signaling (SOCS) proteins such as SOCS3, which interfere with insulin receptor substrate signaling and reduce the expression of essential antioxidant defenses (e.g., catalase), collectively tipping the redox balance toward oxidation and further promoting ROS accumulation. This heightened oxidative and inflammatory state causes cellular damage in insulin-producing β-cells and vascular endothelium and further increases insulin resistance and hyperglycemia, establishing a vicious feed-forward cycle.

### 3.4. Amino Acid Dysregulation and Redox Imbalance

Pregnancy is characterized by enhanced maternal protein turnover, with an increase in proteolysis contributing to a greater availability of circulating amino acids for transplacental transport to support fetal growth [89]. Beyond their role as substrates for protein synthesis, several amino acids and their metabolites act as key metabolic regulators and signaling molecules, which contribute to the pathophysiology of GDM through mechanisms involving redox imbalance, inflammation and insulin resistance. Among these, BCAAs, tryptophan and methionine are most strongly implicated in the pathogenesis of GDM. BCAAs, particularly leucine, serve as potent activators of the mTORC1 signaling pathway. Leucine and methionine activate mTORC1 through Rag GTPase-mediated lysosomal localization, facilitating its interaction with Rheb-GTP. Activated mTORC1 subsequently phosphorylates downstream target S6K1. Persistent activation of S6K1 induces serine phosphorylation of IRS-1, promoting its degradation and impairing insulin signaling. This disrupts the normal propagation of the insulin signal via the PI3K–AKT pathway, and translocation of GLUT4 receptors to the plasma membrane [90].

mTORC1 signaling also promotes IFN-γ production by T cells and natural killer (NK) cells while simultaneously upregulating the expression of its receptor, Interferon Gamma Receptor 1 (IFNGR1) [90] as represented in the Figure 2. IFNGR1 functions as part of a heterodimer with constitutively expressed Interferon Gamma Receptor 2 (IFNGR2) [91]. Binding of IFN-γ to this IFNGR1/IFNGR2 complex induces a conformational change that activates JAK1, associated with IFNGR1, and JAK2, associated with IFNGR2. Activated JAK1 phosphorylates specific tyrosine residues on IFNGR1, creating docking sites for STAT1. STAT1 is then recruited to the phosphorylated receptor, phosphorylated by JAKs, dimerized, and translocated to the nucleus [90]. Once in the nucleus, STAT1 promotes the transcription of suppressor of cytokine signaling-1 (SOCS1). SOCS1 then inhibits IRS-1 phosphorylation at tyrosine residues, thereby impairing the insulin-stimulated PI3K/AKT signaling pathway, resulting in inhibition of GLUT4 translocation to the plasma membrane [92,93].

Additionally, impaired activity of enzymes involved in BCAA oxidation, such as the branched-chain α-ketoacid dehydrogenase (BCKDH) complex, promotes the buildup of intermediates, which disrupt mitochondrial function and enhance the production of ROS. The resulting mitochondrial stress and oxidative imbalance further reinforce mTORC1 hyperactivation and impair insulin signaling, thereby establishing a feed-forward cycle that perpetuates metabolic dysfunction and insulin resistance [94]. In pregnancy, these alterations may be further amplified by pregnancy-associated hormonal changes, including elevated placental hormones and inflammatory mediators, as well as increased maternal nutrient flux [95]. Tryptophan metabolites, including kynurenine, serotonin, and melatonin, further modulate the IFNGR1–JAK1–STAT1 signaling axis [96]. Since metabolites of the kynurenine pathway, such as 3-hydroxykynurenine, 3-hydroxyanthranilic acid and kynurenic acid, play important roles in fetal physiological adaptation during pregnancy, it is conceivable that tryptophan is preferentially shunted toward the kynurenine pathway [97]. Kynurenine can also activate the aryl hydrocarbon receptor (AhR) in T cells and NK cells, promoting Th1 differentiation and thereby stimulating IFN-γ transcription [98].

Similarly, maternal lactogens markedly induce islet serotonin production and secretion, which acts in an autocrine/paracrine manner through the 5-hydroxytryptamine receptor 2B (5-HT2B) receptor to increase total β-cell mass and support glucose-dependent insulin secretion [99]. However, serotonin can also stimulate mTORC1 through GPCR-mediated PI3K/AKT signaling, promoting metabolic reprogramming for robust IFN-γ production [100]. Acting through Melatonin Receptor 1A (MTNR1A or MT1) and Melatonin Receptor 1B (MTNR1B or MT2) receptors on pancreatic β-cells, it modulates insulin secretion and is upregulated during pregnancy to support β-cell function and glucose homeostasis [101]. Similarly, elevated melatonin derived from increased serotonin metabolism can promote IFN-γ release by activating G-protein-coupled MT1 and MT2 receptors on immune cells, triggering downstream PI3K/AKT and MAPK signaling pathways [102].

## 4. Effect on Insulin Signaling and β-Cell Activity

### 4.1. Oxidative Damage and Insulin Resistance Mechanisms

ROS can disrupt insulin signaling pathways through multiple interconnected mechanisms. Under normal conditions, insulin binds to its receptor tyrosine kinase (RTK), inducing receptor dimerization and autophosphorylation of its own tyrosine residues, thereby activating its kinase domain. The activated RTK then phosphorylates tyrosine residues on IRS-1 proteins, creating docking sites for other signaling molecules (like PI3K, Growth factor receptor-bound protein 2 (Grb2)) via their Src Homology 2 (SH2) domains. PI3K generates PIP_3_. PIP_3_ in turn activates protein kinase B and also helps in GLUT4 translocation to the plasma membrane for glucose uptake. Oxidative stress interferes with this cascade, mainly via aberrant serine/threonine phosphorylation of IRS proteins. Stress-sensitive kinases, including JNK, p38 MAPK, IKK, mTOR, and PKC isoforms, phosphorylate IRS-1 proteins on specific serine residues, impairing their interaction with the insulin receptor. This modification promotes IRS-1 dissociation and targets it for proteasome-dependent degradation, ultimately reducing insulin signaling [103,104]. It also allows protein tyrosine phosphatase 1B to dephosphorylate the receptor, thereby downregulating insulin signaling and contributing to insulin resistance [105].

In normo-glycemic conditions, IRS-1 is predominantly associated with the low-density microsomal (LDM) fraction (which is a distinct, buoyant fraction of intracellular membrane vesicles obtained through subcellular fractionation), rather than the plasma membrane. This LDM fraction also contains intracellular vesicles and membrane networks, including the intracellular pool of GLUT4-containing vesicles [106]. Upon insulin stimulation, IRS-1 is phosphorylated by the activated insulin receptor within the LDM fraction. This phosphorylation promotes the recruitment of the regulatory subunit (p85) of PI3K to IRS-1, enabling the formation of an active IRS-1–PI3K (p85/p110) complex specifically in the LDM compartment. This localized complex is essential for propagating downstream insulin signaling, particularly for the mobilization and translocation of GLUT4-containing vesicles to the plasma membrane, which facilitates glucose uptake. However, under conditions of oxidative stress, this finely regulated process is disrupted. AKT-mediated phosphorylation of IRS-1/2 leads to their dissociation from the membrane, followed by their degradation or mislocalization away from the LDM fraction. As a result, the insulin-stimulated redistribution of IRS-1 and PI3K between the cytosol and LDM is impaired. This prevents effective activation of PI3K within the LDM, thereby hindering the proper trafficking of GLUT4 storage vesicles to the cell surface and attenuating insulin signaling. Sustained oxidative stress exacerbates these defects by further impairing GLUT4 translocation, ultimately leading to insulin resistance. In such conditions, residual glucose uptake becomes increasingly dependent on GLUT1, a constitutively expressed glucose transporter. At the transcriptional level, oxidative stress also downregulates GLUT4 expression, compounding the defect in glucose handling. Additionally, oxidative stress disrupts mitochondrial function, creating a vicious cycle that amplifies reactive oxygen species production and impairs cellular energy metabolism. Moreover, the accumulation of lipid intermediates such as DAGs and ceramides under oxidative stress conditions further aggravates insulin resistance. These lipid metabolites activate PKC isoforms, which induce serine phosphorylation of IRS proteins, thereby inhibiting their normal function in insulin signaling. Collectively, these molecular alterations converge to impair insulin action and contribute to the development and progression of insulin resistance [107].

### 4.2. Effects on Pancreatic Β-Cell Oxidative Vulnerability

Pancreatic β-cells are extremely susceptible to oxidative damage because of their intrinsically low levels of antioxidant enzymes. Compared to hepatocytes, they have roughly 50% of superoxide dismutase activity and only 5% of hydrogen peroxide-scavenging activity, including catalase and glutathione peroxidase, with catalase expression being nearly absent. This limited antioxidant capacity makes β-cells especially vulnerable to ROS, particularly under hyperglycemia, which increases mitochondrial ROS production [108,109]. In GDM conditions, chronic hyperglycemia and insulin resistance place extraordinary demands on β-cells, as glucose-stimulated insulin secretion increases mitochondrial oxidative phosphorylation and ROS generation. Excess ROS cause lipid peroxidation, protein oxidation, and DNA damage, triggering β-cell apoptosis through multiple pathways [73]. ROS oxidize cardiolipin in the inner mitochondrial membrane, releasing cytochrome c to activate apoptosome formation and caspase cascades. Oxidative stress also opens mitochondrial permeability transition pores, releasing pro-apoptotic factors, and impairs key transcription factors, including pancreatic and duodenal homeobox 1 (Pdx1) and V-maf musculoaponeurotic fibrosarcoma oncogene homolog (MafA), reducing insulin gene expression and β-cell function. Activation of uncoupling protein-2 by ROS decreases the ATP/ADP ratio, further impairing glucose-stimulated insulin secretion. Additionally, hyperglycemia and ROS can induce an iron-dependent cell death pathway known as ferroptosis. Ferroptosis leads to lipid peroxidation, mitochondrial shrinkage, and ultimately the loss of β-cells. Cumulatively, these effects reduce the mass and function of β-cells in GDM, which further impairs glucose-stimulated insulin secretion and limits β-cell proliferation. Maternal hyperglycemia develops when β-cells cannot meet pregnancy-induced insulin demands, which further creates a vicious cycle of oxidative stress and progressive β-cell dysfunction [110,111].

## 5. Consequences of Oxidative Stress

Oxidative-stress-driven impairments in insulin signaling in GDM have effects that reach well beyond maternal glucose regulation but also contribute to vascular abnormalities and metabolic programming across generations. The impairment of insulin signaling cascades in maternal tissues results in inadequate glucose clearance that leads to maternal hyperglycemia. This hyperglycemic state in turn generates additional ROS through mitochondrial overproduction, AGEs formation, and activation of pro-oxidant enzymes, which creates a self-perpetuating cycle of oxidative damage and metabolic dysfunction [112,113]. Oxidative stress in GDM also disrupts key mechanisms of glucose regulation by damaging pancreatic β-cells and promoting insulin resistance. β-cells are susceptible to damage due to low antioxidant defenses. On the other hand, oxidative stress reduces their insulin secretion by causing apoptosis, mitochondrial dysfunction, blocking ATP-sensitive potassium channels, and suppressing β-cell regeneration. At the same time, oxidative stress also impairs insulin signaling in peripheral tissues. ROS activate serine kinases that reduce insulin receptor phosphorylation and insulin sensitivity. They also reduce GLUT4 levels in muscle and fat, which decreases the glucose uptake capacity [10,14,103].

Endothelial dysfunction represents another major consequence of oxidative stress in GDM, with profound consequences for both maternal and fetal health. ROS impairs NO bioavailability, due to which it compromises endothelium-dependent vasodilation and increases the vascular tone [80,114]. Simultaneously, oxidative stress induces the expression of cell adhesion molecules and chemotactic factors that promote leukocyte adhesion, transmigration, and vascular inflammation. These vascular impairments can contribute to the increased risk of preeclampsia, placental insufficiency, and cardiovascular complications in females with GDM [114,115]. Placentas from women with GDM demonstrate markedly elevated production of oxidative stress markers, including 8-isoprostane, malondialdehyde, 4-hydroxynonenal, protein carbonyls, and xanthine oxidase, as compared to placentas from normoglycemic pregnant women. When the oxidative stress exceeds placental antioxidant defense mechanisms, the damage is not only limited to placenta, but it also affects maternal circulation and distant tissues, which is reflected by elevated serum levels of lipid peroxidation products and ROS, along with reduced total antioxidant capacity [10,116]. Also, the oxidative stress, when combined with endothelial dysfunction, disrupts normal placental functions such as nutrient transport and vascular regulation. As a result, placentas of women with GDM display structural abnormalities like increased weight, fibrinoid necrosis, vascular lesions, altered villous morphology, and impaired expression of nutrient transporters that are involved in balanced delivery of oxygen and nutrients to the fetus. These abnormalities in nutrient transporters permit excessive transfer of glucose and lipids, which creates a metabolically harmful intrauterine environment [117,118].

Altered fetal metabolic programming through epigenetic mechanisms is the most profound and enduring consequence of oxidative stress in GDM. The developmental origins of health and disease framework speculates that adverse intrauterine exposure during the critical period of fetal development can permanently alter tissue architecture and its physiological function, which predisposes offspring to disease in later life. Oxidative stress serves as a key mediator of such programming effects by inducing epigenetic modifications including DNA methylation, histone modifications, and microRNA expression changes in fetal tissues [112,119]. Exposure to maternal hyperglycemia and oxidative stress in utero has been shown to alter DNA methylation patterns in multiple fetal organs, including the liver, pancreas, adipose tissue, heart, and hypothalamus. These epigenetic changes affect genes involved in glucose metabolism, insulin signaling, lipid metabolism, adipogenesis, and appetite regulation. For instance, oxidative-stress-induced hypermethylation of pancreatic and duodenal homeobox-1 and other key β-cell transcription factors in fetal pancreatic tissue may contribute to reduced β-cell mass and impaired insulin secretion in offspring, predisposing them to glucose intolerance and diabetes in adulthood [113,120,121].

Maternal GDM, on the other hand, also promotes offspring adiposity by altering early fat tissue development and raising long-term metabolic risk. It drives mesenchymal stem cells in adipose tissue to favor adipocyte rather than bone or muscle cell formation through increased proliferator-activated receptor-γ (PPAR-γ) activity, leading to more fat cells and greater lipid storage. Consequently, children exposed in utero have higher body mass index (BMI), body fat percentage, waist-to-height ratio, and a greater risk of metabolic syndrome, independent of maternal pre-pregnancy BMI. This risk is amplified when GDM is combined with maternal obesity; for example, girls born to mothers with both conditions have markedly elevated BMI and body fat. These effects are more strongly reflected in central adiposity measures than in BMI alone, pointing to a shift toward metabolic dysfunction rather than just increased body weight [19,121,122].

Beyond metabolic consequences, emerging evidence suggests that maternal GDM and associated oxidative stress may affect neurodevelopmental outcomes in offspring. Children exposed to GDM during pregnancy have been found to have a 42% increased risk of developing autism spectrum disorder, potentially mediated through oxidative-stress-induced alterations in fetal brain development and neurotransmitter systems [121].

## 6. Biomarkers of Oxidative Stress in GDM

### 6.1. Commonly Studied Oxidative Markers

#### 6.1.1. Malondialdehyde

Malondialdehyde is one of the most established biomarkers of lipid peroxidation in GDM, as it forms during oxidative breakdown of polyunsaturated fatty acids and reliably reflects oxidative stress levels. Women with GDM consistently show higher serum malondialdehyde than healthy pregnant women, often even before hyperglycemia is detected, and longitudinal studies confirm that these elevations appear in early trimesters and persist until metabolic control is achieved [123,124]. These levels correlate positively with fasting plasma glucose, Hba1c, maternal body mass index, and age, linking malondialdehyde not only to poor glycemic control but also to obesity and aging as contributors to oxidative stress. Reported concentrations range from around 8.3 ± 1.19 Nmol/mL to higher values depending on disease severity and gestational stage [125]. Elevated malondialdehyde is also found in placental tissue from GDM pregnancies, suggesting local oxidative damage that may affect fetal development. Supporting this, a meta-analysis showed significantly increased maternal plasma and placental Malondialdehyde Levels in GDM, with a standardized mean difference of 1.99 (95% CI: 1.51–2.48), confirming consistent findings across populations. However, with adequate glycemic control and treatment, malondialdehyde levels can return to near-normal by late pregnancy, indicating that GDM-related oxidative stress is at least partly reversible [33].

#### 6.1.2. 8-Hydroxy-2′-Deoxyguanosine (8-OHdG)

8-OHdG, also known as 8-oxo-7,8-dihydro-2′-deoxyguanosine, is the most common and sensitive biomarker of oxidative DNA damage. It forms when hydroxyl radicals attack the C8 position of deoxyguanosine in DNA, and if not repaired before replication, it can cause GC-to-TA mutations. In GDM, hyperglycemia generates ROS through redox-dependent activation of serine/threonine kinase pathways, promoting 8-OHdG formation while suppressing the repair enzyme 8-oxoguanine-DNA glycosylase [126]. This molecular process has clinical relevance, as prospective studies show that higher maternal urinary 8-OHdG levels in early pregnancy predict later development of GDM. Women in the highest quartile of urinary 8-OHdG at 10–13 weeks of gestation exhibited a 3.79-fold higher risk of developing GDM compared to those in the lowest quartile, after adjusting for maternal age, pre-pregnancy body mass index, race/ethnicity, parity, and family history of diabetes. This predictive capacity is particularly pronounced among overweight women, with those having both elevated 8-OHdG and body mass index ≥ 25 kg/m^2^ demonstrating a 6.36-fold increased risk [127].

#### 6.1.3. Protein Carbonyls

Protein carbonylation represents an irreversible oxidative modification that serves as a hallmark of protein oxidative damage in GDM. Carbonyl groups are introduced into proteins through multiple mechanisms: direct oxidation of amino acid side chains (particularly proline, arginine, lysine, and threonine), or secondary reactions of cysteine, histidine, and lysine residues with reactive carbonyl compounds such as aldehydes and ketones derived from lipid peroxidation or glycation/glycoxidation reactions [128]. Studies showed that women with GDM have significantly higher protein carbonyl levels than healthy pregnant women, with elevated concentrations observed particularly at 16–20 and 32–36 weeks of gestation, suggesting fluctuating oxidative stress as pregnancy progresses. Although no significant differences are seen at 24–28 weeks or between GDM alone and GDM with preeclampsia, this may be because protein carbonyls act as early precursors of broader carbonyl stress, affecting both conditions similarly [129]. In placental tissue from women with GDM, protein carbonyl content is significantly high as compared to normal placentas (*p* < 0.004), which reflects the intense oxidative environment within the diabetic placenta. The accumulation of carbonylated proteins promotes further ROS generation, damages cellular components, triggers apoptosis, and contributes to placental dysfunction and adverse pregnancy outcomes [129].

#### 6.1.4. Antioxidant Enzyme Activities

The enzymatic antioxidant defense system consists of superoxide dismutase (SOD), catalase (CAT), and glutathione peroxidase (GPx). They constitute the primary cellular protection against ROS-mediated damage. In GDM, the activities of these enzymes exhibit complex and sometimes paradoxical alterations that reflect both compensatory upregulation and oxidative inactivation. Sod represents the first line of defense against ROS, which catalyzes the dismutation of superoxide radicals to hydrogen peroxide and molecular oxygen. In women with GDM, Sod activity demonstrates tissue-specific and temporal variations. A meta-analysis of oxidative stress biomarkers revealed a significant decrease in Sod levels in maternal plasma from women with GDM as compared to controls, with a standardized mean difference of −2.80 (95% CI: −5.23, −0.36) [33]. However, placental Sod activity has been reported to increase in some GDM cases, which possibly reflects a compensatory response to elevated placental oxidative stress. Interestingly, erythrocyte Sod activity is elevated in pregnancy with insulin-dependent diabetes as compared to normal physiological pregnancy, yet placental tissue Sod activity significantly decreases. This suggests differential regulatory mechanisms across various compartments [130,131].

GPx is a selenium-dependent enzyme that catalyzes the reduction of hydrogen peroxide and organic hydroperoxides to water and corresponding alcohols using reduced glutathione as an electron donor. It exhibits reduced activity in early pregnancy among women who subsequently develop GDM. Serum GPx activity in diabetic women is significantly lower during the first trimester, with mean values progressively increasing during gestation as a compensatory adaptation. However, in the third trimester and the postpartum period, GPx activity remains compromised, particularly in women with GDM complicated by preeclampsia [124]. A significant negative correlation exists between serum malondialdehyde concentration and GPx activity in the second and third trimesters, which confirms that antioxidant depletion runs parallel with intensification of lipid peroxidation [123]. Notably, erythrocyte GPx activity is elevated in pregnancy with GDM as compared to normal pregnancy, while placental GPx activity increases substantially, contrasting with the decreased placental SOD [131].

Catalase is another enzyme that directly decomposes hydrogen peroxide to water and oxygen. It demonstrates elevated activity in some GDM populations, particularly those with concurrent preeclampsia, alongside paradoxically reduced SOD and GPx activities. This imbalanced antioxidant enzyme profile disrupts redox homeostasis during the third trimester, which exacerbates lipid peroxidation as evidenced by the glutathione-to-glutathione-disulfide ratio (GSH: GSSG) [132]. Plasma catalase levels measured in GDM cohorts have shown increases at 16–20 weeks and 32–36 weeks of gestation, which suggests temporal activation patterns that may reflect disease severity or compensatory mechanisms [124].

## 7. Novel and Emerging Biomarkers

### 7.1. Cell-Free Mitochondrial DNA

As ROS overwhelm feto-maternal antioxidant defenses, the resulting trophoblast apoptosis and mitochondrial fragmentation shed genetic material in the blood that serves as a real-time “Liquid Biopsy” of cellular damage. While total cell-free DNA (cfDNA) reflects systemic inflammatory strain, circulating cell-free mitochondrial DNA (ccf-mtDNA) is particularly significant; due to its bacterial ancestry and lack of protective histones, it acts as a pro-inflammatory damage-associated molecular pattern (DAMP) that triggers TLR activation. This in turn recruits the nucleotide-binding domain, leucine-rich repeat family, pyrin domain containing 3 (Nlrp3) inflammasome in placental and adipose tissues, triggering the release of pro-inflammatory cytokines such as IL-1β and IL-18 [124,125]. This cascade drives the low-grade systemic inflammation and insulin resistance characteristic of GDM. Sahu and Abraham demonstrated that elevated ccf-mtDNA levels in the first trimester are associated with the subsequent development of GDM. These increases were independent of maternal age and fasting blood sugar, suggesting that ccf-mtDNA may serve as a sensitive early predictor beyond conventional risk factors [133].

### 7.2. MicroRNAs (MiRNAs)

MiRNAs are short non-coding RNA molecules that control gene expression post-transcriptionally and have emerged as capable biomarkers and mechanistic mediators of GDM. Circulating miRNAs packaged within exosomes exhibit remarkable stability in biological fluids and can cross biological barriers, which makes them ideal candidates for non-invasive biomarker discovery [134]. In placental tissues from GDM women, mir-125b and mir-144 show consistent dysregulation in both circulating exosomes and placental tissue. Specifically, mir-125b is downregulated while mir-144 is upregulated in GDM, with mir-144 concentration in circulating exosomes negatively correlating with pre-pregnancy BMI and delivery weight and positively correlating with 1-h glucose levels during oral glucose tolerance testing (OGTT). These studies proposed that mir-144 may serve as a quantitative biomarker reflecting metabolic disturbances [134,135].

### 7.3. Advanced Oxidation Protein Products (AOPPs)

AOPPs represent a novel class of oxidized proteins formed through the action of chlorinated oxidants, particularly hypochlorous acid and chloramines produced by activated neutrophils and monocytes during oxidative and inflammatory processes. These protein modifications serve as reliable markers of oxidative damage mediated by inflammatory pathways [136]. In GDM, AOPPs are significantly elevated in maternal plasma at multiple gestational time points. A prospective nested case–control study demonstrated that plasma levels of advanced oxidation protein products were elevated at 16–20 weeks and 32–36 weeks of gestation in women with GDM compared to controls but showed no significant difference at 24–28 weeks, suggesting temporal fluctuations potentially linked to disease progression or compensatory mechanisms. These elevated levels were observed before the clinical diagnosis of GDM, which indicates that the protein oxidation occurs early in disease pathogenesis and may contribute to both the development and progression of the condition [129]. Recent investigations have revealed that women with both GDM and preeclampsia exhibit the highest AOPPs levels compared to those with GDM alone or healthy controls. AOPPs measured in maternal serum demonstrate positive correlations with Hba1c and markers of vascular dysfunction. Furthermore, AOPPs buildup is emerging as a therapeutic target for preventing and delaying the development of microvascular complications in diabetes, as medications targeting AOPP-RAGE interactions can reduce binding and mitigate downstream pathological effects [137].

### 7.4. Placental F2-Isoprostanes

The placenta serves as A critical interface between maternal and fetal circulations and represents a primary source of oxidative stress in GDM. Placental tissue from GDM pregnancies exhibits a constellation of oxidative stress biomarkers that reflect both the intensity of oxidative damage and the adequacy of compensatory antioxidant responses [118]. F2-isoprostanes, particularly 8-Isoprostane (8-Epi-Prostaglandin F2α), constitute sensitive and stable biomarkers of lipid peroxidation produced through free radical-catalyzed peroxidation of arachidonic acid independent of cyclooxygenase enzymes. Placental release of 8-Isoprostane is about twice as high in women with GDM compared with women who have healthy pregnancies, with this elevation being highly significant (*p* < 0.001). Elevated 8-Isoprostane Possesses biological activity, capable of inducing vasoconstriction in placental vessels, modulating platelet function, and exhibiting teratogenic potential [129,130,138].

### 7.5. Total Antioxidant Capacity (TAC)

TAC represents an integrated measure of the cumulative action of all antioxidants present in biological fluids, including both enzymatic antioxidants (Sod, Cat, Gpx) and non-enzymatic antioxidants (Vitamins C and E, β-carotene, glutathione, uric acid, albumin, and bilirubin). This composite biomarker provides a holistic assessment of the body’s capacity to neutralize ROS and reflects the balance between oxidative stress and antioxidant defenses [139]. A cross-sectional study found significantly higher levels of salivary TAC in women with GDM compared to non-diabetic pregnant women, which suggests increased antioxidant mobilization to counteract oxidative stress. Conversely, serum TAC was found to be significantly decreased in GDM Women as compared to normal pregnancy, which indicates antioxidant depletion overwhelmed by excessive ROS production1 [40]. A recent prospective study revealed markedly and autonomously high serum TAC at the end of the first trimester in women who subsequently established GDM later in pregnancy. This elevated level suggests an early compensatory response where increased free radical levels trigger enhanced endogenous antioxidant production before hyperglycemia becomes clinically apparent. A significant negative correlation exists between oxidative stress index and TAC in the GDM group, which confirms the defensive role of antioxidants in modulating oxidative burden [140].

## 8. Targeting Oxidative Stress: Current and Emerging Strategies

### 8.1. Evidence from Antioxidant Therapies

Antioxidant intake can be a helpful strategy in clinical care for women who have been diagnosed with GDM. Among the most extensively studied antioxidant vitamins, Vitamin C and Vitamin E have demonstrated variable efficacy in GDM prevention and management. Observational studies revealed a strong inverse relationship between dietary Vitamin C intake and GDM risk. Women consuming < 70 mg/day of Vitamin C exhibit a significantly increased risk of GDM compared to those with higher intakes. And consumption beyond 200 mg/day confers a 32% lower risk of GDM as compared to normal intakes [141]. A recent case–control study demonstrated that Vitamin C supplementation alleviates the risk of GDM by modulating inflammatory cytokines and oxidative stress biomarkers. Participants in the highest tertile of Vitamin C supplementation exhibited significantly lower fasting blood glucose levels and improved insulin sensitivity as compared to those in the lowest tertile [142]. Oral Vitamin C doses of 500 to 1000 mg/day are considered potentially effective, safe, and affordable for many people with diabetes, demonstrating improvements in glycemic control and reductions in oxidative stress markers [143]. Polyphenols such as resveratrol have also demonstrated significant therapeutic potential in GDM. Resveratrol exerts beneficial effects through activation of sirtuin 1, which subsequently stimulates AMP-activated protein kinase to improve mitochondrial biogenesis and function, which ultimately enhances insulin sensitivity, lowers oxidative damage, and regulates metabolic homeostasis [144]. Pilot studies have reported improvements in glucose levels, total cholesterol, low-density lipoprotein, and triglycerides when resveratrol is combined with D-chiro inositol and myo-inositol [144,145]. Coenzyme Q10 (CoQ10) is another endogenous lipid-soluble antioxidant that is essential for the proper functioning of the mitochondrial electron transport chain. CoQ10 helps maintain healthy mitochondrial activity, supports ovarian and placental function, and may lower the risk of obesity-related problems like preeclampsia and miscarriage [146]. CoQ10 can help improve both the number and quality of oocytes in aging ovaries. It may also support healthier oxidative balance in follicular fluid, strengthen mitochondrial activity in oocytes, and raise the chances of pregnancy [147]. A comprehensive systematic review and meta-analysis revealed significant improvements in insulin resistance markers after antioxidant supplementation, such as zinc, selenium, and alpha lipoic acid. They concluded that antioxidant supplementation is a beneficial adjunct therapy to current dietary management for women with GDM [148].

### 8.2. Pharmacological Modulation

Metformin lowers circulating glucose levels and improves insulin sensitivity. Additionally, metformin inhibits glucagon signaling and hepatic glucose production by suppressing adenylate cyclase activity. Collectively, these actions contribute to a marked reduction in chronic hyperglycemia. Current clinical guidelines issued by the World Health Organization (WHO), the International Diabetes Federation (IDF), the American College of Obstetricians and Gynecologists (ACOG), and the American Diabetes Association (ADA) recommend metformin as a second-line pharmacological option for the treatment of GDM when insulin is either not feasible, unavailable, or declined by the patient [149]. Metformin exhibits multiple effects on mitochondrial activity and oxidative stress. A recent study investigated the effects of metformin on placental metabolism, as well as mitochondrial content and function, in syncytiotrophoblast (STB) derived from placentas of pregnancies carrying male and female fetuses. The findings demonstrated that the effects of metformin are influenced by both maternal metabolic status and fetal sex. Treatment with metformin (≥1 mM) enhanced glycolysis in STB from lean women; however, an increase in glycolytic capacity was observed only in placental tissue from pregnancies with female fetuses. Additionally, metformin had minimal impact on superoxide production in placental tissue from male fetuses, whereas placental tissue from female fetuses, irrespective of maternal BMI status, exhibited a dose-dependent reduction in superoxide generation [150]. Beyond direct mitochondrial effects, metformin also modulates placental epigenetic regulation, which influences the expression of genes involved in nutrient transport, inflammation, and oxidative stress responses [151]. Long-term follow-up studies examining intrauterine metformin exposure to offspring have documented mixed findings regarding metabolic outcomes, growth trajectories, and developmental programming. This highlights the need for continued surveillance of children born to metformin-treated mothers [152]. Glyburide is a second-generation sulfonylurea that also demonstrated effectiveness in the management of GDM. Owing to its prolonged duration of action, glyburide is typically administered once daily [149]. Pregnancy significantly influences the pharmacokinetics of glyburide. Pregnant women tend to exhibit lower plasma concentrations of the drug, largely due to enhanced renal and hepatic clearance. Consequently, the pharmacokinetic profile of glyburide is altered in patients with GDM, which may necessitate adjustments in doses to achieve optimal glycemic control [149].

Inositol stereoisomers such as myo-inositol and D-chiro-inositol also function as insulin-sensitizing agents that modulate glucose metabolism and have demonstrated efficacy in GDM prevention. Both myo-inositol and D-chiro-inositol exhibit insulin-mimetic effects in conditions of insulin resistance, which influences tissue-specific glucose metabolism. A comprehensive meta-analysis revealed that myo-inositol supplementation significantly reduced incidence of GDM [153]. In contrast, combinations of myo-inositol with D-chiro-inositol did not demonstrate significant benefits, which potentially reflects suboptimal dosing or ratios. Myo-inositol supplementation also demonstrated significant effects on reducing prematurity rates, although no differences were observed between groups in cesarean section rates, neonatal hypoglycemia, macrosomia, gestational hypertension, preeclampsia, or insulin therapy requirements [153]. Multi-centric randomized trials administering myo-inositol and D-chiro-inositol plus Vitamin D from 12 weeks of gestation demonstrated an improved trend in reducing GDM rates (3.22% in the treatment group versus 5.08% in the vitamin D-alone group), although the difference did not reach statistical significance [154].

Mitoquinone mesylate (MitoQ), a mitochondrial-targeted antioxidant comprising ubiquinone attached to a triphenylphosphonium cation by a 10-carbon alkyl chain, represents a novel therapeutic approach specifically targeting mitochondrial oxidative stress. MitoQ rapidly accumulates within the inner mitochondrial membrane due to the large mitochondrial membrane potential, achieving concentrations several hundred-fold higher than in the cytoplasm, where it is transformed to the active antioxidant ubiquinol for the reduction of mitochondrial ROS [155]. MitoQ demonstrates efficacy in multiple pregnancy complications beyond metabolic disorders. In rodent preeclampsia models, MitoQ alleviated symptoms and improved placental mitochondrial function while restoring pup weight at birth. A recent study in a preeclampsia mouse model demonstrated that MitoQ supplementation attenuated hypertension and proteinuria through dampening Gαq signaling and ROS production [156]. MitoQ is already available in a stable oral form and has been given to people without any serious side effects, positioning it as a reasonable candidate for clinical translation. In skeletal muscle insulin resistance models, MitoQ treatment improved GLUT-4 translocation to the plasma membrane in response to insulin stimulation, demonstrating positive effects on glucose tolerance [157].

## 9. Future Therapeutic Directions

Precision medicine approaches in GDM aim to stratify patients based on individual characteristics, enabling more targeted and effective interventions. Systematic reviews have identified multiple precision markers from routine clinical measurements that enable earlier identification of women requiring escalation of pharmacological therapy. These markers include previous history of GDM, body mass index, blood glucose concentrations at diagnosis, maternal age, parity, family history of diabetes, gestational age at diagnosis, and history of macrosomia in previous pregnancies [157,158]. Women with lower maternal age, nulliparity, lower body mass index, no previous GDM history, lower glycated hemoglobin, lower glucose values from the diagnostic oral glucose tolerance test, no family history of diabetes, and later gestational age at diagnosis are more likely to achieve adequate glycemic control with lifestyle measures alone, whereas those with opposite characteristics are more likely to require pharmacological escalation.

Artificial intelligence (AI), mainly machine learning and deep learning algorithms, has shown strong potential for predicting early GDM risk, supporting continuous glucose monitoring, guiding insulin dosing, and estimating clinical outcomes. Machine learning models use clinical, biochemical, and lifestyle data to enhance precision and efficiency in the management of GDM. The Medición Integrada para la Detección Oportuna (MIDO) AI model achieved high accuracy (70.3%) and sensitivity (83.3%) for identifying women at high risk of developing GDM using eight easily collected variables like age, family history of type 2 diabetes, previous hypertension diagnosis, pregestational body mass index, gestational week, parity, birth weight of the last child, and random capillary glucose [159]. A systematic review of AI use in GDM care included 126 studies, and about 85% of them centered on early GDM prediction. Classical machine learning was dominant, with 86% in which logistic regression and random forest were most frequently employed. While internal validation was common (68%), external validation remained rare (6%), which highlights the need for broader validation efforts before clinical implementation [160].

Advancements in omics technologies like proteomics, metabolomics, and genomics have led to identification of novel biomarkers by revealing molecular pathways involved in GDM progression. A recent review collated biomarkers that are associated with GDM and identified 278 biomarkers across multiple molecular classes, including proteins, cell-free DNA, long non-coding RNAs, microRNAs, and metabolites [158]. Integration of metabolomics with proteomics and clinical risk factors significantly improves GDM prediction models, with refined models achieving areas under the curve of 0.85 as compared to 0.63 for clinical factors alone [161]. Multibiomarkers that combine protein, metabolic, and nucleic acid markers may offer better predictive performance than relying on a single biomarker. However, validated biomarkers and models for predicting GDM risk remain scarce despite substantial research efforts [158]. Many biomarkers that show strong individual associations still lack replication and validation, underscoring the need for consistent evaluation using standardized approaches and multicenter studies. Future research must focus on validating biomarkers and models in large, multiethnic prospective cohorts to determine economic feasibility of biomarker testing in routine early pregnancy screening programs and to explore synergistic combinations of interventions targeting multiple pathophysiological pathways.

## 10. Conclusions

Studies shows that oxidative stress is not just a consequence of high blood sugar but a key driver in the development of GDM. It connects metabolic imbalance, inflammation, insulin resistance, and loss of β-cell function. In a healthy pregnancy, oxidative activity is regulated to support placental growth. However, this balance is disrupted in GDM. Excess ROS overcome antioxidant defenses, especially in the placenta, which triggers mitochondrial dysfunction and several harmful metabolic pathways. These changes weaken insulin signaling, limit glucose uptake, and promote β-cell death, which reduces insulin production. Oxidative stress also appears early in pregnancy and can predict who will later develop GDM, which points to its value as an early risk marker. The fact that GDM can persist even when blood sugar is well managed suggests that treatments targeting oxidative pathways may need to complement standard care. Also, consistent research methods will be essential to turn these findings into clinical tools to reduce long-term metabolic risks for both mother and offspring.

## Data Availability

No new data were created or analyzed in this study. Data sharing is not applicable to this article.

## Abbreviations

5-hydroxytryptamine receptor 2B (5-HT2B); 5-methyltetrahydrofolate (5-methylTHF); 66 kDa Src homology 2 domain-containing protein (p66Shc); A Disintegrin and Metalloproteinase 17 (ADAM17); Activator Protein-1 (AP-1); Advanced Glycation Endproducts (AGEs); Advanced Oxidation Protein Products (AOPP); aryl hydrocarbon receptor (AhR); Branched-Chain Amino Acids (BCAA); branched-chain α-ketoacid dehydrogenase (BCKDH); Carnitine Palmitoyltransferase I (CPT-1); Chronic Low-Grade Inflammation (CLGI); c-Jun N-terminal Kinase (JNK); c-Jun N-terminal kinase (JNK); c-Jun N-terminal Kinase (or c-Jun N-terminal protein kinase) (JNK); Coenzyme A (CoA); Coenzyme Q10 (CoQ10); Damage-Associated Molecular Pattern (DAMP); diacylglycerol (DAG); Dynamin-related protein 1 (Drp1); Endoplasmic Reticulum (ER); Endoplasmic Reticulum (ER); endothelial nitric oxide synthase (eNOS); Flavin adenine dinucleotide (reduced form) (FADH2); Free Fatty Acid Receptor 2 GPR43 (FFAR2); Free Fatty Acid Receptor 3 (GPR41 (FFAR3); G Protein-Coupled Receptor 120 (also known as Free Fatty Acid Receptor 4) (GPR120 (FFAR4)); Gestational diabetes mellitus (GDM); Glucose Transporter type 1(GLUT1); Glucose Transporter type 4 (GLUT4); Glutathione Peroxidase (GPx); glutathione to glutathione disulfide ratio (GSH:GSSG); G-protein-coupled receptor 40 (GPR40); Growth Factor Receptor-Bound Protein 2 (Grb2); GTPase-Activating Protein activity toward Rags 1 (GATOR1); Hemoglobin A1c (or glycated hemoglobin) (HbA1c); Hydrogen peroxide (H_2_O_2_); Hydroxy-2′-Deoxyguanosine (8-OHdG); Insulin Receptor Substrate-1 (IRS-1); Interferon Gamma Receptor 1 (IFNGR1); Interferon Gamma Receptor 2 (IFNGR2); Interferon-gamma (IFN-γ); Interleukin-1 beta (IL-1β); Interleukin-6 (IL-6); IκB kinase (Inhibitor of nuclear factor kappa-B kinase) (IKK); IκB kinase β (IKKβ); Janus Kinase 1/Signal Transducer and Activator of Transcription 1 (JAK1/STAT1); KPTN, ITFG2, C12orf66, and SZT2-containing regulator (KICSTOR); lipopolysaccharide (LPS); low-density microsome (LDM); LPS-binding protein (LBP); Mammalian Target of Rapamycin Complex 1 (mTORC1); Medición Integrada para la Detección Oportuna (Integrated Measurement for Timely Detection) Gestational Diabetes Mellitus artificial intelligence model (MIDO GDM AI Model); Melatonin Receptor 1A (MTNR1A); Melatonin Receptor 1B (MTNR1B); Mitogen-Activated Protein Kinase (MAPK); Mitogen-Activated Protein Kinase (MAPK); Mixed-Lineage Leukemia 1 (MLL1)-dependent Histone H3 Lysine 4 trimethylation (MLL1-dependent H3K4me3); Monocyte Chemoattractant Protein-1 (MCP-1); Myeloid Differentiation Primary Response 88 (MyD88); NADPH oxidase (NOX); Nicotinamide adenine dinucleotide (NAD); Nicotinamide adenine dinucleotide (reduced form) (NADH); Nicotinamide Adenine Dinucleotide Phosphate (reduced form) (NADPH); nitric oxide (NO); NOD-like receptor family pyrin domain-containing 3 (NLRP3); Nuclear Factor kappa-light-chain-enhancer of activated B cells (NF-κB); Nuclear Factor kappa-light-chain-enhancer of activated B cells (p65 subunit/RelA)(NF-κB (p65)); Pancreas/duodenum homeobox protein 1 (also known as Pancreatic and duodenal homeobox factor-1) (PDX1); Peroxisome Proliferator-Activated Receptors (PPARs); peroxynitrite (ONOO^−^); phosphatidylinositol (3,4,5) trisphosphate (PIP3); phosphatidylinositol (3,4,5)-trisphosphate (PIP3); Phosphatidylinositol 3-Kinase/Protein Kinase B (PI3K/Akt); Polyunsaturated Fatty Acids (PUFA); proliferator-activated receptor-γ (PPAR-γ); protein kinase C (PKC); Reactive Oxygen Species (ROS); Receptor for Advanced Glycation Endproducts (RAGE); receptor tyrosine kinase (RTK); Ribosomal protein S6 kinase 1 (S6K1); S-adenosylmethionine sensor upstream of mTORC1 (SAMTOR); S-adenosylmethionine, (SAM); short-chain fatty acids (SCFAs); Skp1–Cullin 1–F-box protein β-Transducin repeat-containing protein E3 ubiquitin ligase (SCF^β TRCP E3); Solute Carrier Family 25 Member 42 (SLC25A42); Sterol Regulatory Element-Binding Protein-1c (SREBP-1c); superoxide dismutase (SOD); suppressor of cytokine signaling-1 (SOCS1); suppressor of cytokine signaling-1 (SOCS1); Suppressor of Cytokine Signaling-3 (SOCS3); Syncytiotrophoblasts (STBs); T helper type 1 cells (Th1); TIR-Domain-Containing Adaptor-Inducing Interferon-β (TRIF); TNF-α Converting Enzyme (TACE); Toll-Like Receptor 4 (TLR4); Toll-like receptor 4 (TLR4)—Myeloid differentiation factor 2 (MD-2) (TLR4–MD-2); Total Antioxidant Capacity (TAC); Tumor necrosis factor-alpha (TNF-α); Unfolded Protein Response (UPR); V-maf musculoaponeurotic fibrosarcoma oncogene homolog A (MAFA).
